# Survey of medical genetic services in Italy: year 2011

**DOI:** 10.1186/s12913-016-1340-7

**Published:** 2016-03-17

**Authors:** Daniela Giardino, Rita Mingarelli, Tiziana Lauretti, Antonio Amoroso, Lidia Larizza, Bruno Dallapiccola

**Affiliations:** Laboratorio di Citogenetica, IRCCS Istituto Auxologico Italiano, Milano, Italy; IRCCS Ospedale Pediatrico Bambino Gesu, Roma, Italy; Dipartimento di Scienze Mediche, Azienda Ospedaliera Città della Salute e della Scienza, Università di Torino, Torino, Italy; Genetica Medica, Dipartimento di Scienze della Salute, Università degli Studi di Milano, Milano, Italy

## Abstract

**Background:**

The aim of this study was to collect information about 2011 genetic activities in Italy, with the purpose of providing guidance to the national health systems in order to improve genetic services.

**Methods:**

A web-based survey was carried out to achieve the information.

**Results:**

Data were collected from 268 macrostructures hosting 517 services and employing 3246 persons. About 295,000 cytogenetic, 35,000 immunogenetic and 263,000 molecular genetic analyses of 902 genes were recorded. Seventy-four percent of the services were accredited with institutional bodies and 57 % were also certified according to ISO 9001 standard. Twenty percent of cytogenetic laboratories had participated in an European External Quality Assessment (EQA) while 44 % participated in a national EQA. Only 28 % of the molecular laboratories had participated in a national Cystic Fibrosis EQA. The percentage of diagnoses confirmed by genetic tests varied among disorders, ranging from 52 % for coeliac disease to 4 % for fragile X syndrome.

**Conclusions:**

This study highlights the need for reorganizing the Italian genetic services network, improving EQA participation and developing national plans for implementing next generation technologies. Concerted effort has to be addressed in the education of the professionals prescribing tests to improve appropriateness and to inform patients, who now have exposure to direct-to-consumer multifactorial genetic testing where clinical utility is unproven.

## Background

The Italian Society of Human Genetics (SIGU) launched in 2012 a survey designed to scrutinize the 2011 activities of the Italian Medical Genetic Services, to compare the results with previous data collections, and to update the national scenario. SIGU had previously carried out six surveys to gather information on cytogenetic and molecular testing and clinical genetics activities, with the aim of providing guidance to the national and regional health systems to improve the organization of genetic services [1, 2].

Here, we report the results of this last study, providing an overview of the changes occurred over a 4-year period, highlighting the need for reviewing and improving the Italian genetic structure network, in line with the current public health requirements.

## Methods

The survey refers to the medical genetic activities performed in Italy from January 1st to December 31st, 2011. This study was carried out by the Bambino Gesù Children Hospital in Rome, in collaboration with Orphanet-Italy, on behalf of SIGU. Ethical approval for this study was granted by SIGU. Data were gathered by an *ad hoc* questionnaire (http://docs.biomedia.net/SIGU/Schema-Censimento_SIGU-2011.pdf) sent to genetic centers hosted by universities, research hospitals (Istituti di Ricovero e Cura a Carattere Scientifico - IRCCS), general hospitals, local health centers (Aziende Sanitarie Locali - ASLs) and commercial laboratories. The enrolled genetic centers were those registered and updated from the 2007 survey, integrated with others recruited through Orphanet database (www.orpha.net) or joining voluntarily to this initiative after learning of it during national scientific conferences or through the SIGU website (www.sigu.net). A verbal informed consent was obtained from participants.

Questions from previous surveys were reiterated and new ones added, in order to achieve information on the activities of immunogenetic laboratories and diagnostic tests becoming available in more recent years (i.e. microarrays). Entered data were based on self-certification. Information was collected online from March to July 2012, using forms available in a dedicated website. Each participating center accessed the website through a private username and password assigned during the registration process. Requested data included total number and type of performed cytogenetic and molecular genetic tests, clinical genetic activities such as the number and type of genetic counseling sessions. Data concerning the personnel attending laboratories and clinical activities were also collected. The quality management system of the responding centers in terms of national accreditation standards and international certifications was investigated, together with national and international External Quality Assessment enrolment. By comparing the number of analyses with the number of diagnoses confirmed by the test results, appropriateness was evaluated for the following entities: achondroplasia, Angelman, DiGeorge, fragile X-mental retardation, Noonan, Prader-Willi and Williams syndromes, ankylosing spondylitis and coeliac diseases. Appropriateness was investigated also for CGH (Comparative Genomic Hybridization) and SNPs (Single Nucleotide Polymorphisms) array tests.

## Results

### Genetic Centers

**Fig. 1:**
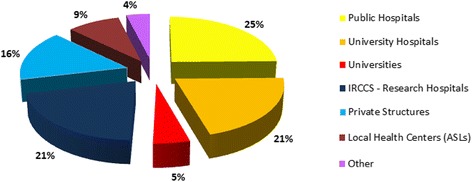
Distribution by affiliation of the 517 services performing genetic activities during 2011 in Italy

**Fig. 2:**
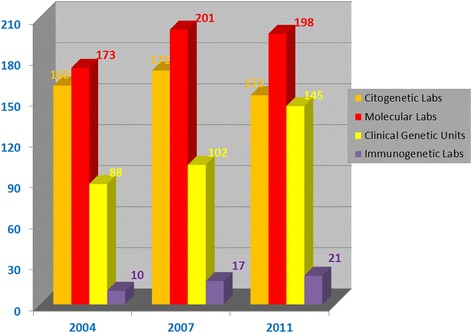
Number of censused structures in the SIGU surveys of 2004, 2007 and 2011

**Fig. 3:**
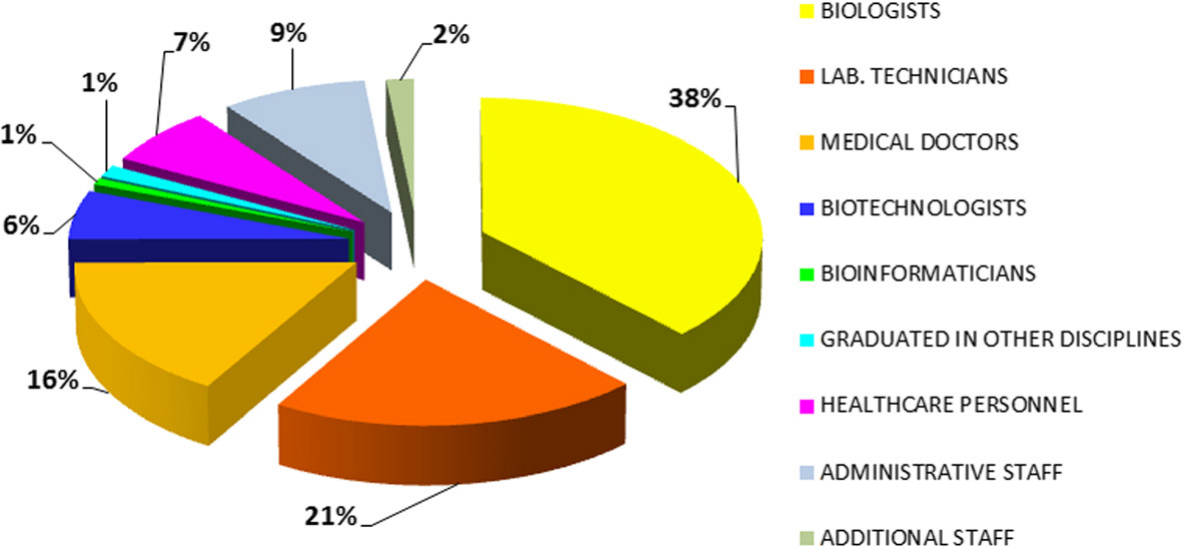
Professional qualifications of the staff employed in the Italian genetic services

Overall, 268 genetic centers, hosting 517 genetic services (genetic testing and counselling) were surveyed (Table 1). 25 % of these services were located in public hospitals, 21 % in university hospitals, 21 % in IRCCS, 16 % in private centers, 9 % in ASLs, 5 % in universities, and 4 % in a different institution (Fig. 1). The genetic units included 145 clinical services and 372 diagnostic laboratories, comprising 153 cytogenetic, 198 molecular, and 21 immunogenetic laboratories (Table 1). By comparing the number of the 2011 censused structures with the previous two surveys, an increase of clinical genetic services appeared evident with a slight decrease in the cytogenetic and molecular genetic laboratories (Fig. 2). The collected data was representative of the genetic services in Italy as a whole. A decreasing gradient of the total number of genetic centers from North to South and to the Islands of Italy was observed in previous studies and was substantiated in the present analysis (Table 1). A total of 3246 persons were employed in the national genetic services, of which 38 % biologists, 21 % technicians, 16 % medical doctors, 6 % biotechnologists, 1 % bioinformaticians, 1 % graduated in different disciplines, 7 % healthcare personnel, 9 % administrative, and 2 % other staff (Fig. 3). Centers that had adopted a Quality Management System are shown in Table 2: 74 % of the services were institutionally accredited by regional bodies. In addition to this institutional accreditation, 57 % of services were also voluntarily certified according to ISO (International Organization for Standardization) 9001 standard, 7 % accredited according to ISO 15189, about 5 % to SIGU and 4 % to JCI (Joint Commission International) standards. Seven out of 21 immunogenetic laboratories had obtained the EFI (European Federation for Immunogenetics) accreditation.

**Table 1:** Geographical distribution of the censused structures

Area	Medical	%	Genetic services								
	genetic centers		CL	%	MGL	%	IGL	%	CGS	%	Total
Northern	142	53	70	46	101	51	8	38	68	47	247
Central	54	20	32	21	45	23	7	33	34	23	118
Southern	45	17	31	20	34	17	5	24	30	21	100
Islander	27	10	20	13	18	9	1	5	13	9	52
Total	268		153		198		21		145		517

*CL* Cytogenetic Laboratories, *MGL* Molecular Genetic Laboratories, *IGL* Immunogenetic Laboratories, *CGS* Clinical Genetic Services, *%*, % of structures located in a specific geographic area out of the total of the censused structures

**Table 2:** Quality assurance by accreditation or certification procedures

Geographical area	Accredited by institutional bodies	Also ISO 9001 certified	Also ISO 15189 accredited	Also ISO 17025 accredited	Also JCI accredited	Also EFI accredited	Also CAC accredited	Also SIGU Accredited
Northern	109	83	13	0	7	4	1	9
Central	42	31	7	1	1	2	0	3
Southern	28	22	0	1	2	0	0	0
Islander	19	17	0	0	0	1	0	1
Total^a^	198	153	20	2	10	7	1	13
%^b^	73,8	57,1	7,4	0,7	3,7	2,6	0,3	4,8

^a^Total number of accredited/certified structures

^b^% of accredited-certified centers out of the total of the censused centers

### Cytogenetic laboratories and activity

Forty-six percent of cytogenetic laboratories were located in the Northern regions, compared with 21 % in Central regions, 20 % in Southern regions, and 11 % in the Islands (Table 1). The total number of cytogenetic laboratories had decreased slightly compared to 2007 (Fig. 2), and the total number of analyses dropped from 312,881 in 2007 to 294,155 in 2011 (Table 3). This diminution occurred in both the constitutional prenatal and postnatal tests, and this decrease may be a consequence of their replacement by the molecular tests. For example, in 2011 a total of 19,908 QF-PCR (Quantitative Fluorescent- Polymerase Chain Reaction) were performed (the 2007 figure is not available) negating the need for full chromosome analysis in some cases. In addition, 1862 prenatal microarray tests were undertaken (compared to 393 in 2007).

**Table 3:** Constitutional and oncological cytogenetic analyses surveyed by SIGU since 2000

	Year				
Type of tissue/analysis	2000	2002	2004	2007	2011
*Prenatal cytogenetic tests*	Number				
Amniotic Fluid	77395	95729	101011	101750	97320
Chorionic villi	9169	15159	18357	25691	25520
Fetal blood	784	808	870	478	383
Spontaneous abortions	2874	5231	6483	8415	5856
Prenatal FISH		8643	20032	26331	2891^a^
Rapid FISH for aneuploidies					4288
Microarray (CGH and SNPs)				393	1862
* Total Prenatal tests*	90222	125570	146753	163058	138120
*Postnatal cytogenetic tests*					
Peripheral blood		65148	81153	8,478	69841
Fibroblasts		493	1258	1184	811
Other		213			502
Postnatal FISH		4776	22999	22653	5819^b^
Microarray (CGH and SNPs)				1443	8290
* Total Postnatal tests*	49696	70630	105410	108758	85263
*Oncological cytogenetic tests*					
Bone marrow		23445	27323	34500	31538
Peripheral blood					2873
Solid tumors		1671			2482
Metaphase ish		8383	4112	6565	4142
Interphase ish					27821
Microarray (CGH and SNPs)					1916
* Total oncological cytogenetic tests*	15948	33499	31435	41065	70772
TOTAL CYTOGENETIC TESTS	155866	229699	283598	312881	294155

^a^FISH for microdeletion syndromes (897), subtelomeric regions (276), extra structurally abnormal chromosomes (599) and rearrangement (1119) characterization. ^b^FISH for microdeletion syndromes (2662), subtelomeric regions (1186), extra structurally abnormal chromosomes (455) and rearrangement (1516) characterization

Cancer cytogenetic tests increased from 2000 onwards with the greater contribution for this escalation being attributable to an increase of FISH (Fluorescent In Situ Hybridization) and ISH (In Situ Hybridization) analyses.

**Fig. 4:**
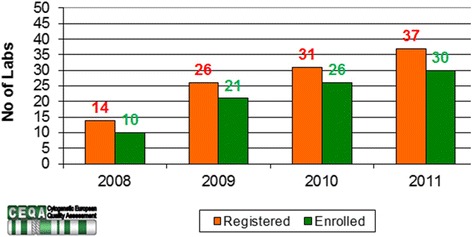
CEQA Registration vs Enrolment (effective participation) of Italian Cytogenetic Laboratories during the last years

One aspect of a Quality Management System is the participation in an accredited external quality assessment (EQA) scheme. Only 30 of 153 censused laboratories (20 %) had taken part in an international accredited Cytogenetic European Quality Assessment (CEQA, now known as CEQAS) (Fig. 4) and 68 (44 %) in the national cytogenetic EQA scheme (personal communication provided by Italian National Centre for rare Diseases –Istituto Superiore di Sanità, Rome, Italy). Figure 4 also shows that the number of laboratories that effectively participated (enrolled) in an international QA (Quality Assessment) is lower than that registered to attend.

### Molecular genetic laboratories and activity

**Fig. 5:**
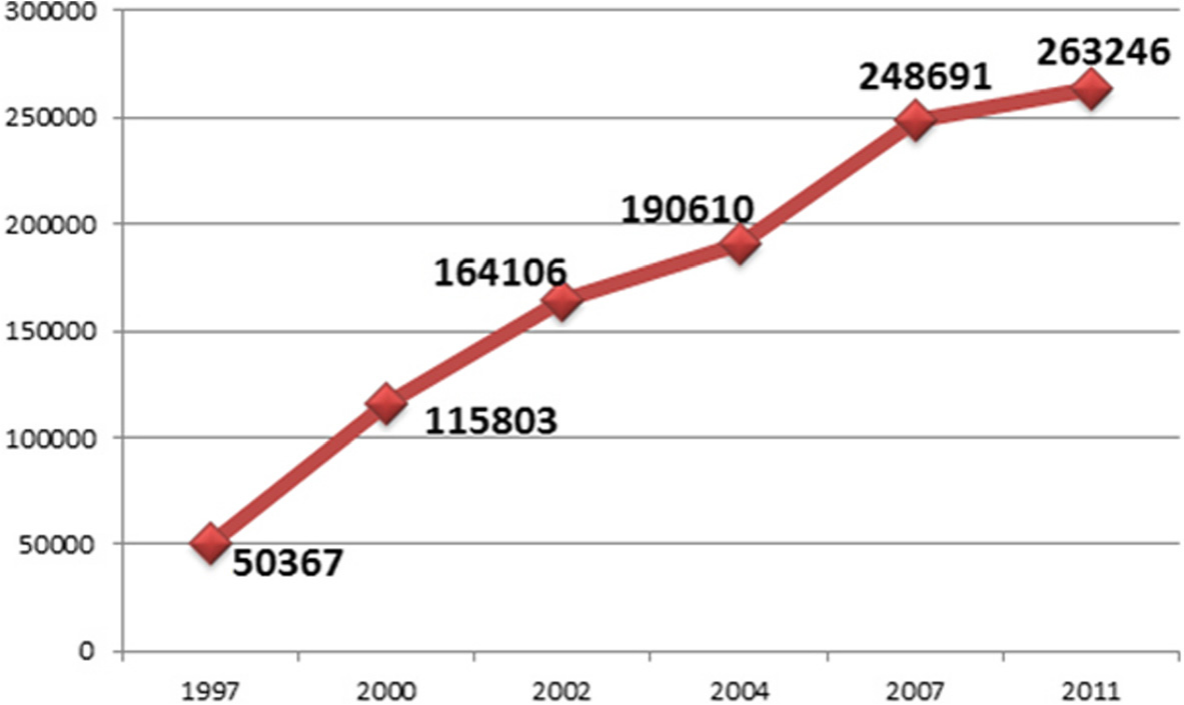
Molecular genetics analyses surveyed by SIGU since 1997

Fifty-one percent of 198 molecular genetic laboratories were located in Northern regions, 23 % in Central regions, 17 % in Southern regions, and 9 % in the Islands (Table 1). The number of surveyed molecular analyses (263,246) increased by 6 %, compared to the 2007 study (248,691) (Fig. 5). The total number of analyzed genes was 902 (Table 4), a figure consistently higher compared with previous 2007 report (556), which is starting to bridge the previous gap between the number of analyzed genes and the number of disease genes amenable for investigation [3]. Seventy percent of the molecular tests (184,679 of 263,246 analyses) included 15 disease genes or group of genes, the analysis of CFTR gene accounting for 21 % of total (Table 5). Tests of susceptibility to common diseases comprised about 64,000 tests (24 % of the total), mainly exploring genes within the thrombophilia cascade and about 18,000 analyses (6 %) of the major histocompatibility complex (HLA), primarily for disease association studies (Table 5). In the 2007 survey, the susceptibility tests accounted for about 37 % of all molecular analyses.

**Table 4:** Disease genes amenable to analysis in Italy and Europe: scenario 2007–2012

	Year		
	2007	2011	2012
Genes tested in Europe (source: Orphanet/EurogenTest)	1500	1812	2179
Genes tested in Italy (source: SIGU survey)	556 (37 %)	902 (50 %)	1042 (48 %)

**Table 5:** Top 15 molecular genetic tests in Italy, year 2011

	Number	Percent
Cystic fibrosis (*CFTR*)	55716	21
Factor V Leiden mutation (*F5*)	24834	9
Coagulation Factor II mutation (*F2*)	20393	8
*MTHFR* Deficit	18526	7
Celiac Disease (*HLA*)	11824	4
Beta thalassemia (*HBB*)	7796	3
Fragile X syndrome (*FRAXA*)	7695	3
Chronic Myeloid Leukaemia (*BCR/ABL*)	7131	3
Rheumatic Diseases (*HLA*)	6431	2
Hemochromatosis (*HFE*)	5088	2
Neurosensory deafness (*GJB6*)	4895	2
Duchenne/Becker muscular dystrophy (DMD)	3823	1
Breast cancer (*BRCA1/2*)	3821	1
Acute Myeloid Leukaemia	3579	1
Y chromosome microdeletion (*AZF*)	3127	1
Total	184679	70

Only 55 of 198 molecular genetic laboratories (28 %) participated in either a national or international cystic fibrosis EQA schemes. It is likely that not all the 198 censused laboratories offered genetic testing for cystic fibrosis, but the majority did, although the participation in EQA was lower than desirable.

**Fig. 6:**
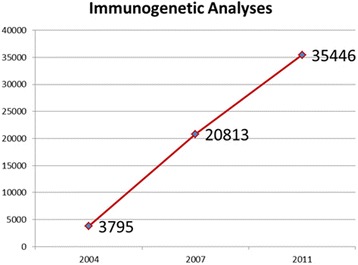
Increase of immunogenetic analyses in Italy since 2004

A total of 35,446 molecular genetic analyses had been performed by 21 immunogenetic laboratories in 2011, indicating a sharp and constant increase during the last 12 years (Fig. 6).

### Clinical genetic services and activity

**Fig. 7:**
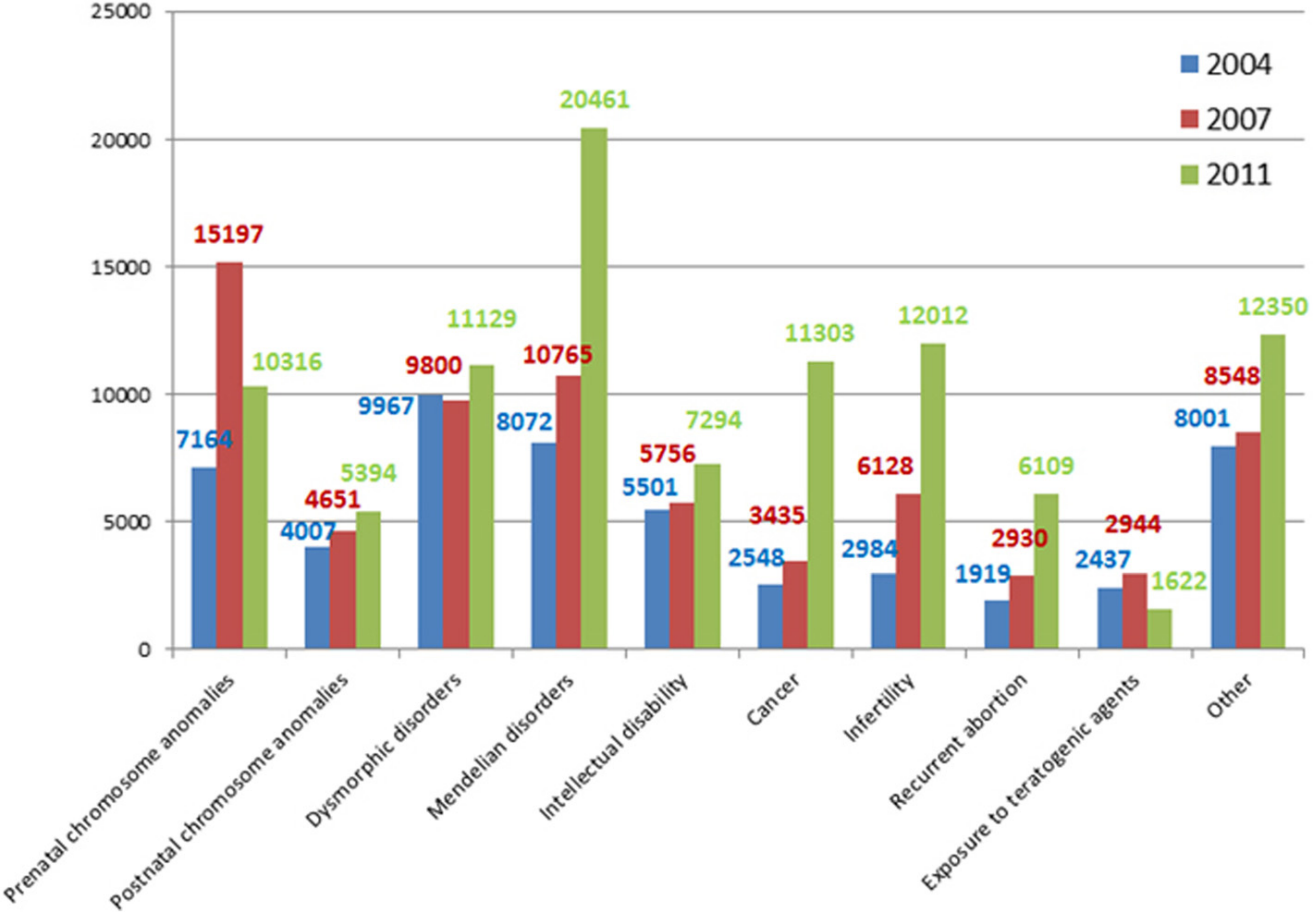
2004-2011 trend in demand for genetic counseling in Italy

Forty-seven percent of 145 clinical genetic services were located in Northern regions, 23 % in Central regions, and 21 % in Southern/Island regions (Table 1). A total of 100,001 genetic counseling sessions were performed in 2011, mainly for single gene diseases (20 %), chromosome anomalies detected at prenatal or postnatal testing (15 %), infertility (12 %), cancer (11 %), dysmorphic syndromes (11 %), and intellectual disability disorders (7 %) (Table 6). The 2004–2011 trend of genetic counseling activity is shown in Fig. 7. The total number of registered genetic counseling sessions with respect to the laboratory diagnostic activities was quite low, only 12 % (11 % in 2007) of all genetic analyses had been accompanied by pre-test or post-test counseling.

**Table 6:** Reason for requesting genetic counselling in 2004–2011 period

	2004	2007	2011
Prenatal chromosome anomalies	7164 (13 %)	15197 (21 %)	10316 (10 %)
Postnatal chromosome anomalies	4007 (7 %)	4651 (6 %)	5394 (5 %)
Dysmorphic disorders	9967 (18 %)	9800 (14 %)	11129 (11 %)
Mendelian disorders	8072 (15 %)	10765 (15 %)	20461 (20 %)
Intellectual disability	5501 (10 %)	5756 (8 %)	7294 (7 %)
Cancer	2548 (5 %)	3435 (5 %)	11303 (11 %)
Infertility	2984 (5 %)	6128 (8 %)	12012 (12 %)
Recurrent abortion	1919 (4 %)	2930 (4 %)	6109 (6 %)
Exposure to teratogenic agents	2437 (4 %)	2944 (4 %)	1622 (2 %)
Other	8001 (15 %)	8548 (12 %)	12350 (12 %)
Total	54604	72161	100001

%, % of reason for demanding genetic counselling out of the total of the requests

### Appropriateness of the tests

The appropriateness of genetic testing was assessed by investigating the congruence between the clinical diagnosis and the test results in nine disorders (Table 7), selected on the basis of their easily recognizable clinical phenotype and the significant number of evaluated cases. Clinical diagnosis was substantiated in 52 % of patients with celiac disease, 15 % with ankylosing spondylitis, 23 % with Williams syndrome (3 % in 2007), 33 % with DiGeorge/Velocardiofacial syndrome (3 % in 2007), 4 % with fragile-X syndrome, 7 % with Angelman syndrome (9 % in 2007), 8 % with Prader-Willi syndrome (18 % in 2007), and 45 % with achondroplasia (36 % in 2007).

**Table 7:** Appropriateness of the requests for genetic testing in seven rare disorders and 2007–2011 trend

Disorder	No. of clinically evaluated individuals	No. of cases confirmed by genetic testing	% of positive test (2011)	% of positive test (2007)	Trend from last survey
Williams Syndrome	610	149	24	3	++
DiGeorge/Velocardiofacial Syndrome	2260	736	33	3	++
Fragile X Syndrome	8240	353	4	4	=
Achondroplasia	186	84	45	36	+
Noonan Syndrome	968	267	28	NE	
Angelman Syndrome	713	49	7	9	-
Prader-Willi Syndrome	927	78	8	18	- -
Ankylosing spondylitis	2826	418	15	NE	
Celiac diseases	6748	3488	52	NE	

*NE* not evaluated

++ significant improvement

+ improvement

= no variations

- worsening

-- significant worsening

Worthy of note was the 4 % figure of the confirmed fragile X syndrome cases. Fifteen percent of CGH-array analyses (1383 out of 9355 tests) disclosed pathogenic Copy Number Variations (CNVs), including 407 deletions resulting in known deletion syndromes.

## Discussion

The survey of the Italian medical genetic centers in year 2011 was carried out on the behalf of SIGU. This study aimed at providing indications to the national and regional public health systems, in agreement with the Regional Health System request to rationalize and integrate the public genetic laboratories under the pressure of the spending review and the need to improve efficiency. Indeed, the national health system is fully supporting the cost of genetic testing requested by the specialists, with some limitations for prenatal testing in women under the age of 35 years and/or without any specific indication. Accordingly to previous reviews, the number of genetic services was exceeding that required against the approximately 60,000,000 total inhabitants. Indeed the Minister of Health and the Italian Regions suggested that the maximums should be 1,000,000 inhabitants for every clinical genetics facility between 500,000 and 1,000,000 inhabitants for every cytogenetic laboratory and not less than 3,000,000 inhabitants for every molecular genetic laboratory (http://www.salute.gov.it/imgs/c_17_pubblicazioni_908_allegato.pdf). The total number of genetic services in Table 1 clearly attests an oversize in all kinds of services.

As shown in this report, the coverage of the Italian territory by genetic testing facilities is one of the highest in Europe. In addition there is not equal distribution of the genetic services across the country, mainly attributable to economic reasons, since both public and private genetic services initially began in the rich regions of Northern Italy, which also are the most densely inhabited [2]. To date, only a few Italian regions have implemented strategic plans for reducing the number of centers through a coordination of their activities [4].

The SIGU 2011 survey evidenced a slightly lower number of genetic services compared to the 2007 data and confirmed that more than 50 % of all genetic services were located in Northern regions. A total of 3246 health professionals worked in these services, 44 % of which were biologists or biotechnologists as compared to only 21 % of technicians, an Italian anomaly contrasting the trend observed in other European countries and mainly attributable to the availability of the former to accept scholarships.

In the last decade guidelines regulating the activities of clinical and laboratory genetic services were jointly developed by the Ministry of Health and the Italian Regions, establishing roles and functions of genetic services [1]. According to National and Regional Health System, public Medical Genetics Services must operate within a Quality Management System, accredited by institutional bodies. Quality is defined by a number of requirements, which are not limited to organizational, professional, technical, procedural and communication elements. A significant improvement in the adoption of Quality Management Systems was registered in 2011, with respect to 2007. Indeed, 74 % of genetics structures turned out to be institutionally accredited, 57 % to be also voluntarily certified according to ISO 9001 standard (28 % in 2007) and a few voluntarily accredited according to ISO 15189. About 5 % of genetic services voluntarily accredited according to the quality standard for genetic structures elaborated by SIGU (www.sigu.net). The Italian state of quality systems within genetic testing services is similar to the European overview provided by Berwouts et al. [5]. Again, a North to South gradient was registered, with the majority of the accredited/certified services being located in Northern regions.

Collected data, showed that only 20 % of cytogenetic laboratories participated in the accredited CEQAS (formerly CEQA) and 44 % in the Istituto Superiore di Sanità (ISS, EQA national provider) schemes. Only a minority of the molecular laboratories stated they participated in an EQA scheme for cystic fibrosis. The participation in EQA is an established tool for improving quality, educating laboratory staff and directing laboratories towards best practices. National rules establish that medical laboratories shall participate in EQA scheme in order to be institutionally accredited, but appropriate checks to verify the participation of the genetic laboratories in EQA are only occasionally carried out by responsible bodies.

As shown in this report, the coverage of the Italian territory by genetic testing facilities is one of the highest in Europe. Table 4 shows that the 50 % of genetic tests available in Europe at time of survey were available also in Italy (902 genes investigated in 2011 compared to the 556 disease genes in 2007). Data collected by Orphanet-Italy referring to year 2012 figure showed an additional increase, with 1107 investigated disease genes. This positive trend places Italy at the fourth position in Europe, after Germany, Spain, and France [3]. In the case of tests not available in Italy, disease-gene analysis is performed using cross-border facilities. According to the Kääriäinen European survey [6] including also data from Italy, about the 3 % of genetic analyses in Europe are performed cross-border.

The need for a revision of the national molecular diagnostic network and for a diversification of the offer are supported by the results of the present survey, showing that testing of 15 disease-genes only accounted for 70 % of all molecular analyses in 2011. In addition, the demand of molecular tests has increased by a figure of 20 % from 2007 onwards, and cytogenetic analyses has reached a plateau of around 300,000 tests per year. Compared with the results registered by the 2007 census, the request of molecularly testing the susceptibility to common disorders and complex phenotypes was not increased, the analysis of thrombophilia cascade genes and HLA accounting for about 30 % of all molecular tests.

The request of constitutional prenatal and postnatal chromosome analyses has decreased in recent years. In particular, the number of genetic tests in amniocytes, which had been stable between 2004–2007, decreased in the years preceding the present survey, while the number of test on chorionic villi, increased by about 2500 per year in 2007–2011, stabilizing at around 25,500. It is expected that the decreasing trend of invasive prenatal tests will be further corroborated in the coming years, due to introduction of Non-Invasive Prenatal Testing (NIPT) into the diagnostic set.

The reduction of constitutional postnatal cytogenetic tests appears mainly related to the decrease of FISH analyses, which was expanded in the early 2000s, in parallel with the increased availability of commercial probes testing for cryptic deletions and supernumerary markers. A similar trend was also seen prenatally, with a consistent decrease of FISH tests. Between 2007–2011, FISH analyses had been progressively substituted by microarrays testing, as shown by the 8290 postnatal and 1862 prenatal tests (18 % of the total) in 2011, compared to 1443 and 393 respectively in 2007. The reduction in FISH prenatal tests was related also to the widespread use of QF-PCR for detecting major aneuploidies.

By contrast, oncological cytogenetic tests had increased by 58 %, mainly as a consequence of the wider introduction of FISH interphase analysis in genetic centers as documented by Table 3. One possible reason is the adoption of interphase FISH for detecting cryptic chromosome rearrangements in neoplastic disorders as recommended by the international guidelines.

As to previous concerns on the appropriateness of genetic testing requested by referring clinical geneticists, the 2011 results show an increase in the confirmed diagnoses for DiGeorge/Velocardiofacial and Williams syndromes, and a significant decrease for Prader-Willi syndrome compared to 2007 (see Table 7).

Only 12 % of all genetic analyses were accompanied by genetic counseling, in contrast with international [7] and national recommendations [8, 9]. Inappropriate replacement of the patient communication session by professionals of contiguous fields could account for the poor detection rate in these syndromes.

Since 2011 no substantial changes have been introduced in the Italian public genetic diagnostic field. In fact, the more recently developed tests (NGS: Next Generation Sequencing; NIPT) have not yet entered in the diagnostics setting and remain restricted to a research application. Public healthcare in Italy is now administrated on a regional basis and a concerted effort must be made to review and rationalize the network of genetic services nationally, taking into account the results of this present survey. In fact, this study provides a real and detailed representation about the logistic of the genetic centers, the number of performed genetic tests, the quality of provided services, the appropriateness of genetic test requests and the number of employees. Within this setting, a number of recommendations can be given for improving genetic services in Italy, which is at present oversized and nonharmonious. Some priority issues are emerging, including management of quality systems in terms of accreditation, certification, and EQA participations; accessibility of pre-test and post-test genetic counseling; the need of increasing availability of genetic testing for rare diseases; the development of national plans and strategies for implementing next generation technologies and advising on the clinical utility of genetic testing for complex disorders. The quality standard for genetic structures elaborated by SIGU may be adopted by a national competent body responsible for the specific accreditation and quality assurance of the genetic services. Another challenge, focused by Battista et al. [4], refers to the need of reconfiguring the professional roles and responsibilities. An additional effort concerns the education and training of those prescribing genetic tests, which should be utilized as a valuable support to good clinical practice and evidence based medicine. A parallel commitment must be to inform citizens, nowadays exposed to an ever more present and available direct-to-consumergenetic testing which has little proven clinical utility.

## Conclusions

This study provides a real and detailed representation about the logistics of the genetic centers operating in Italy, the quality of their provided services, the number of performed genetic tests, and the number of employees. There are too many genetic services given the population size of Italy and they are not equally distributed across the country. Quality assurance of the genetic services may be improved through a specific accreditation standard. Good clinical practice and evidence based medicine may be implemented through the education and training of the people involved in the prescriptions of genetic tests. The finding that 70 % of all molecular analyses performed in 2011 was related to only 15 disease-genes, strongly supports the need for a revision of the national molecular diagnostic network. All together, these results provide information useful for improving genetic services in Italy.

## Abbreviations

ASLs: Aziende Sanitarie Locali
CEQA: Cytogenetics European Quality Assessment
CEQAS: Cytogenetics European Quality Assessment Service
CGH: comparative genomic hybridization
CNVs: copy number variations
EFI: European Federation for Immunogenetics
EQA: external quality assessment
FISH: fluorescent in situ hybridization
IRCCS: Istituti di Ricovero e Cura a Carattere Scientifico
ISH: in situ hybridization
ISO: International Organization for Standardization
ISS: Istituto Superiore di Sanità
JCI: Joint Commission International
NGS: next generation sequencing
NIPT: non-invasive prenatal testing
QF-PCR: quantitative fluorescent. Polymerase Chain Reaction
SIGU: Italian Society of Human Genetics
SNPs: single nucleotide polymorphisms
